# Robertsonian Translocations: An Overview of 872 Robertsonian Translocations Identified in a Diagnostic Laboratory in China

**DOI:** 10.1371/journal.pone.0122647

**Published:** 2015-05-01

**Authors:** Wei-Wei Zhao, Menghua Wu, Fan Chen, Shuai Jiang, Hui Su, Jianfen Liang, Chunhua Deng, Chaohui Hu, Shihui Yu

**Affiliations:** 1 KingMed Genome Diagnostic Laboratory, Guangzhou, China; 2 Department of Laboratory Medicine, University of Washington School of Medicine and Seattle Children’s Hospital, Seattle, Washington, United States of America; Mayo Clinic, UNITED STATES

## Abstract

Robertsonian translocations (ROBs) have an estimated incidence rate of 1/1000 births, making this type of rearrangement the most common structural chromosomal abnormalities seen in the general population. In this study, we reports 872 cases of ROBs from 205,001 specimens karyotyped postnatally in a single accredited laboratory in China, including 583 balanced ROBs, 264 unbalanced ROBs, 9 mosaic ROBs, and 18 complex ROBs. Ninety-three percent of the balanced ROBs observed were adults with infertility, miscarriage, or offspring(s) with known chromosomal abnormalities. Significant excess of females were found to be carriers of balanced ROBs with an adjusted male/female ratio of 0.77. Ninety-eight percent of the unbalanced ROBs observed were children with variable referral reasons. Almost all of the unbalanced ROBs involved chromosome 21 except a single ROB with [46,XX,der(13;14),+13] identified in a newborn girl with multiple congenital anomalies. Multiple novel ROB karyotypes were reported in this report. This study represents the largest collections of ROBs in Chinese population.

## Introduction

Robertsonian translocations (ROBs) are chromosomal rearrangements that result from the fusion of the entire long arms of two acrocentric chromosomes. The karyotype of a balanced ROB shows only 45 chromosomes in which the translocation chromosome contains the two complete long arms of the two acrocentric chromosomes involved while the short arms of the two translocated chromosomes are lost. ROBs have an estimated incidence rate of 1/1000 births, making this type of rearrangements the most common structural chromosomal abnormalities seen in the general population [1–3]. Although all human acrocentric chromosomes (chromosomes 13, 14, 15, 21, and 22) are capable of participating in ROB formation, producing 5 types of homologous ROBs and 10 types of heterologous ROBs, the distribution of different ROBs in the general population is nonrandom, with der(13q14q) and der(14q21q) constituting ∼85% of all ROBs and all other types of ROBs constituting the remaining ∼15% of these translocations. The majority of heterologous ROBs are inherited from a carrier parent and the minority of them form *de novo* mainly in the stage of meiosis I of oogenesis whereas almost all homologous ROBs form *de novo* mitotically [4,5]. It was proposed that the high prevalence of ROBs is because of the similarities of the DNA sequences shared by the short arms of acrocentric chromosomes which confer susceptibility to chromosome rearrangement [3]. The predominance of the der(13q14q) and der(14q21q) may be specifically due to homologous but inverted segments in these pairs of chromosomes that facilitate crossover and recombination, while the variable breakpoints in the uncommon translocations occur randomly [6,7]. Most carriers of a balanced ROB, both heterologous and homologous, do not display an obvious phenotype and remain undetected until they attempt to reproduce. However, a heterologous ROB carrier can produce offsprings with either a normal karyotype or a balanced ROB through alternate segregation of meiosis or produce unbalanced gametes through adjacent segregation of meiosis leading to increased risk of infertility, spontaneous miscarriage, offsprings with unbalanced translocations, and uniparental disomy (UPD) or UPD-related imprinting disorders if chromosomes 14 and 15 are involved. In contrast, a homologous ROB carrier can only produce unbalanced gametes (either disomic or nullisomic), when fertilized with a normal gamete, leading to the formation of conceptuses with either trisomy or monosomy. Occasionally a homologous ROB carrier could have a phenotypically normal child by postzygotic trisomy rescue mechanism in which the free chromosome from the normal gamete is lost at a very early mitosis. Of the 5 acrocentric chromosomes, unbalanced der(21;21) is the most common chromosomal category after standard trisomy 21 resulting in Down syndrome (DS), conceptuses with an unbalanced der(13) are occasionally viable whereas none of the other unbalanced possibilities (trisomies 14, 15, and 22, and any of the monosomies) are viable.

There are about 16 million newborns in China in 2011, of which 5.6% are affected by different types of birth defects (Report on birth defects in China, 2012. http://www.gov.cn/gzdt/att/att/site1/20120912/1c6f6506c7f811bacf9301.pdf). Chromosomal abnormalities including trisomies are considered to be one of the most important causes of the birth defects. Different from the birth defects surveillance systems in developed countries where almost all babies with suspected DS and other chromosomal abnormalities—related disorders will be karyotyped to confirm the diagnosis [4,8–10], only a small number of these types of patients have been karyotyped in China due to limited access to chromosomal analyses. Given the fact that approximately 5% of individuals with DS in Caucasians are due to a translocation involving chromosome 21, and about 95% of them are ROBs [2], it is invaluable in many ways to know the prevalence of both balanced and unbalanced ROBs in Chinese populations. We noticed that a couple of studies reported some data involving ROBs in small sample sizes of Chinese populations [11,12]. In the current study, we shared our large collection of both balanced and unbalanced ROBs identified postnatally in a single accredited laboratory in China.

## Materials and Methods

From Jan., 2011 to June, 2014, we successfully performed karyotype analyses for 205,001 human postnatal specimens in our CAP and ISO 15189 accredited central laboratory in Guangzhou, Guangdong province, China, including 98,686 (48.14%) males, 105,995 (51.70%) females, and 320 (0.015%) individuals with ambiguous or unknown gender. The male/female ratio in this cohort for known individuals is 0.93 (98,686 / 105,995). Referral reasons for testing varied greatly. The majority of children referred for testing involved one or more of the following clinical findings: developmental delay (DD), autism, dysmorphic features (DF), seizures, or multiple congenital anomalies (MCA). The majority of adults referred for testing mainly because of infertility, miscarriage, or offspring(s) with known chromosomal abnormalities. These specimens came from all over the mainland of China except several provinces in Western China. The proportions of specimens from individuals with Chinese ethnic minorities are not clearly documented. Given the facts that more than 94% of Chinese in mainland China are Chinese Han and very few of the tested specimens included in this study were from the Western areas of China where the ethnic minorities are highly concentrated, ROBs included in this study are not categorized according to their ethnics. All adult participants in this study have provided their written consent by the participants or their caretakers. We obtained written consent for all minors/children enrolled in this study from their parents or guardians. The institutional review board of KingMed Genome Diagnostic Laboratory approved this study protocol.

Standard chromosomal analyses with trypsin-Giemsa banding were performed on routinely cultured peripheral blood lymphocytes for all ROBs. Twenty metaphases were microscopically analyzed for non-mosaic cells and sixty metaphases were analyzed for mosaic cells. Nomenclatures were assigned for each ROB based on the international system for human cytogenetic nomenclature (ISCN 2013). The International System for Human Cytogenetics Nomenclature (ISCN) 2013 guidelines have standard nomenclature to describe a ROB using abbreviated “der” or “rob” in lower ROB. In this article, a shorthand description for ROBs [e.g., der(13;14), der(21;21)] has been used throughout although the majority of homologous acrocentric rearrangements are likely to be isochromosomes.

## Results

Of the 205,001 specimens karyotyped in this study, 872 were identified to have ROBs. These ROBs are categorized into 4 groups: (1) 583 balanced ROBs (Table 1A–1C and S1 Fig, S3 Fig, S4a Fig, S5–S7 Figs, S9–S11a Figs, S12 Fig and S15 Fig), (2) 264 unbalanced ROBs (Table 2 and S2a Fig, S4B Fig, S8a Fig, S11B Fig, S13 Fig), (3) 9 mosaic ROBs in which either an additional normal or abnormal cell population was found in addition to the cell population carrying a ROB (Table 3) and (4) 18 complex ROBs in which multiple genomic abnormalities were present (Table 3 and S2c Fig, S2d Fig, S8b Fig, S14 Fig). Two ROBs are grouped into both categories 3 and 4 because they are both mosaic and complex.

**Table 1:** Total Cases of balanced Robertsonian translocations (ROBs) detected in this study.

Subtypes	der(13;13)			der(13;14)			der(13;15)			der(13;21)			der(13;22)			der(14;14)			der(14;15)			der(14;21)		
	**T**	**M**	**F**	**T**	**M**	**F**	**T**	**M**	**F**	**T**	**M**	**F**	**T**	**M**	**F**	**T**	**M**	**F**	**T**	**M**	**F**	**T**	**M**	**F**
**Number**	**5**	**2**	**3**	**344**	**164**	**180**	**24**	**9**	**15**	**16**	**7**	**9**	**14**	**4**	**10**	**4**	**1**	**3**	**23**	**5**	**18**	**86**	**35**	**51**
**Percentages (%)**	**0.86**	**0.34**	**0.51**	**59.01**	**28.13**	**30.87**	**4.12**	**1.54**	**2.57**	**2.74**	**1.20**	**1.54**	**2.40**	**0.69**	**1.72**	**0.69**	**0.17**	**0.51**	**3.95**	**0.86**	**3.09**	**14.75**	**6.00**	**8.75**
**M/F Ratio**	**0.67**			**0.91**			**0.60**			**0.78**			**0.40**			**0.33**			**0.28**			**0.69**		
Adjusted M/F Ratio*	**0.72**			**0.98**			**0.65**			**0.84**			**0.43**			**0.36**			**0.30**			**0.74**		
**Table 1B. Balanced Robertsonian translocations (ROBs) detected in adults.**																								
**Subtypes**	**der(13;13)**			**der(13;14)**			**der(13;15)**			**der(13;21)**			**der(13;22)**			**der(14;14)**			**der(14;15)**			**der(14;21)**		
	**T**	**M**	**F**	**T**	**M**	**F**	**T**	**M**	**F**	**T**	**M**	**F**	**T**	**M**	**F**	**T**	**M**	**F**	**T**	**M**	**F**	**T**	**M**	**F**
**Number**	**5**	**2**	**3**	**323**	**151**	**172**	**23**	**9**	**14**	**13**	**5**	**8**	**12**	**4**	**8**	**4**	**1**	**3**	**22**	**5**	**17**	**80**	**33**	**47**
**Percentages (%)**	**0.92**	**0.37**	**0.55**	**59.38**	**27.76**	**31.62**	**4.23**	**1.65**	**2.57**	**2.39**	**0.92**	**1.47**	**2.21**	**0.74**	**1.47**	**0.74**	**0.18**	**0.55**	**4.04**	**0.92**	**3.13**	**14.71**	**6.07**	**8.64**
**M/F Ratio**	**0.72**			**0.94**			**0.69**			**0.67**			**0.54**			**0.36**			**0.32**			**0.75**		
Adjusted M/F Ratio*	**0.77**			**1.02**			**0.74**			**0.72**			**0.58**			**0.39**			**0.34**			**0.81**		
**Chi Square Test**	**NT**			**P>0.5**			**P>0.1**			**P>0.1**			**P>0.1**			**NT**			**P<0.02**			**P>0.1**		
**Table 1C. Balanced Robertsonian translocations (ROBs) detected in children.**																								
**Subtypes**	**der(13;13)**			**der(13;14)**			**der(13;15)**			**der(13;21)**			**der(13;22)**			**der(14;14)**			**der(14;15)**			**der(14;21)**		
	**T**	**M**	**F**	**T**	**M**	**F**	**T**	**M**	**F**	**T**	**M**	**F**	**T**	**M**	**F**	**T**	**M**	**F**	**T**	**M**	**F**	**T**	**M**	**F**
**Number**	**0**	**0**	**0**	**21**	**13**	**8**	**1**	**0**	**1**	**3**	**2**	**1**	**2**	**0**	**2**	**0**	**0**	**0**	**1**	**0**	**1**	**6**	**2**	**4**
**Percentages (%)**	**0.00**	**0.00**	**0.00**	**53.85**	**33.33**	**20.51**	**2.56**	**0.00**	**2.56**	**7.69**	**5.13**	**2.56**	**5.13**	**0.00**	**5.13**	**0.00**	**0.00**	**0.00**	**2.56**	**0.00**	**2.56**	**15.38**	**5.13**	**10.26**
**M/F Ratio**	**0.00**			**1.63**			**0.00**			**2.00**			**0.00**			**0.00**			**0.00**			**0.50**		
Adjusted M/F Ratio*	**0.00**			**1.75**			**0.00**			**2.15**			**0.00**			**0.00**			**0.00**			**0.54**		
**Chi Square Test**	**NT**			**P>0.1**			**NT**			**NT**			**NT**			**NT**			**NT**			**NT**		

Subtypes	der(14;22)			der(15;15)			der(15;21)			der(15;22)			der(21;21)			der(21;22)			der(22;22)			All types of ROBs		
	**T**	**M**	**F**	**T**	**M**	**F**	**T**	**M**	**F**	**T**	**M**	**F**	**T**	**M**	**F**	**T**	**M**	**F**	**T**	**M**	**F**	**T**	**M**	**F**
**Number**	**14**	**6**	**8**	**5**	**2**	**3**	**16**	**6**	**10**	**9**	**1**	**8**	**6**	**0**	**6**	**14**	**2**	**12**	**3**	**2**	**1**	**583**	**246**	**337**
**Percentages (%)**	**2.40**	**1.03**	**1.37**	**0.86**	**0.34**	**0.51**	**2.74**	**1.03**	**1.72**	**1.54**	**0.17**	**1.37**	**1.03**	**0.00**	**1.03**	**2.40**	**0.34**	**2.06**	**0.51**	**0.34**	**0.17**	**100**	**42.20**	**57.80**
**M/F Ratio**	**0.75**			**0.67**			**0.60**			**0.13**			**0.00**			**0.17**			**2.00**			**0.73**		
Adjusted M/F Ratio*	**0.81**			**0.72**			**0.65**			**0.13**			**0.00**			**0.18**			**2.15**			**0.78**		
**Table 1B. Balanced Robertsonian translocations (ROBs) detected in adults.**																								
**Subtypes**	**der(14;22)**			**der(15;15)**			**der(15;21)**			**der(15;22)**			**der(21;21)**			**der(21;22)**			**der(22;22)**			**All types of ROBs**		
	**T**	**M**	**F**	**T**	**M**	**F**	**T**	**M**	**F**	**T**	**M**	**F**	**T**	**M**	**F**	**T**	**M**	**F**	**T**	**M**	**F**	**T**	**M**	**F**
**Number**	**14**	**6**	**8**	**2**	**0**	**2**	**14**	**6**	**8**	**9**	**1**	**8**	**6**	**0**	**6**	**14**	**2**	**12**	**3**	**2**	**1**	**544**	**227**	**317**
**Percentages (%)**	**2.57**	**1.10**	**1.47**	**0.37**	**0.00**	**0.37**	**2.57**	**1.10**	**1.47**	**1.65**	**0.18**	**1.47**	**1.10**	**0.00**	**1.10**	**2.57**	**0.37**	**2.21**	**0.55**	**0.37**	**0.18**	**100.00**	**41.73**	**58.27**
**M/F Ratio**	**0.81**			**0.00**			**0.81**			**0.13**			**0.00**			**0.18**			**2.00**			**0.72**		
Adjusted M/F Ratio*	**0.87**			**0.00**			**0.87**			**0.14**			**0.00**			**0.19**			**2.15**			**0.77**		
**Chi Square Test**	**P>0.5**			**NT**			**P>0.5**			**P<0.001**			**NT**			**P<0.01**			**NT**			**P<0.01**		
**Table 1C. Balanced Robertsonian translocations (ROBs) detected in children.**																								
**Subtypes**	**der(14;22)**			**der(15;15)**			**der(15;21)**			**der(15;22)**			**der(21;21)**			**der(21;22)**			**der(22;22)**			**All types of ROBs**		
	**T**	**M**	**F**	**T**	**M**	**F**	**T**	**M**	**F**	**T**	**M**	**F**	**T**	**M**	**F**	**T**	**M**	**F**	**T**	**M**	**F**	**T**	**M**	**F**
**Number**	**0**	**0**	**0**	**3**	**2**	**1**	**2**	**0**	**2**	**0**	**0**	**0**	**0**	**0**	**0**	**0**	**0**	**0**	**0**	**0**	**0**	**39**	**19**	**20**
**Percentages (%)**	**0.00**	**0.00**	**0.00**	**7.69**	**5.13**	**2.56**	**5.13**	**0.00**	**5.13**	**0.00**	**0.00**	**0.00**	**0.00**	**0.00**	**0.00**	**0.00**	**0.00**	**0.00**	**0.00**	**0.00**	**0.00**	**100.00**	**48.72**	**51.28**
**M/F Ratio**	**0.00**			**2.00**			**0.00**			**0.00**			**0.00**			**0.00**			**0.00**			**0.95**		
Adjusted M/F Ratio*	**0.00**			**2.15**			**0.00**			**0.00**			**0.00**			**0.00**			**0.00**			**1.02**		
**Chi Square Test**	**NT**			**NT**			**NT**			**NT**			**NT**			**NT**			**NT**			**P>0.5**		

**Table 2:** Unbalanced Robertsonian translocations (ROBs) detected in Children.

Subtypes	46,der(13;14),+13			46,der(13;21),+21			46,der(14;21),+21			46,der(15;21),+21			46,der(21;21)			46,der(21;22),+21			All types of ROBs		
**Number**	**T**	**M**	**F**	**T**	**M**	**F**	**T**	**M**	**F**	**T**	**M**	**F**	**T**	**M**	**F**	**T**	**M**	**F**	**T**	**M**	**F**
	**1**	**0**	**1**	**17**	**6**	**11**	**108**	**57**	**49**	**6**	**4**	**2**	**123**	**64**	**59**	**6**	**4**	**2**	**259**	**135**	**124**
**Percentage**	**0.39**	**0.00**	**0.39**	**6.56**	**2.32**	**4.25**	**41.70**	**22.01**	**18.92**	**2.32**	**1.54**	**0.77**	**47.49**	**24.71**	**22.78**	**2.32**	**1.54**	**0.77**	**100.00**	**52.12**	**47.88**
**M/F ratio**	**0.00**			**0.55**			**1.16**			**2.00**			**1.08**			**2.00**			**1.09**		
M/F ratio*	**0.00**			**0.59**			**1.25**			**2.15**			**1.17**			**2.15**			**1.17**		

Notes: T: total; M: male; F: female;

*based on the M/F ratio of the 98,686 males and 105,995 females performed by karyotype analysis.

**Table 3:** Total cases of mosaic/complex Robertsonian translocations (ROBs) detected in this study.

Case	Sex	Age	Karyotype	der(13;14)	der(13;21)	der(14;21)	der(21;21)	der(21;22)	der(22;22)
**1**	**F**	**0**	**46,XX,der(14;21)(q10;q10),+18**			**x**			
**2**	**F**	**0**	**46,XX,der(13;14)(q10;q10),+21**	**x**					
**3**	**F**	**0**	**46,XX,der(13;14)(q10;q10),+21**	**x**					
**4**	**M**	**0**	**mos 46,XY,der(21;21)(q10;q10)[40]/45,XY,der(13;21)(q10;q10)[20]**		**x**		**x**		
**5**	**M**	**0**	**46,XY,der(13;14)(q10;q10),+21**	**x**					
**4**	**M**	**0**	**46,XY,der(13;14)(q10;q10),+21**	**x**					
**7**	**M**	**0**	**46,XY,der(13;14)(q10;q10),+21**	**x**					
**8**	**M**	**0**	**46,XY,der(13;14)(q10;q10),+21**	**x**					
**9**	**M**	**1**	**46,XY,der(13;14)(q10;q10),+21**	**x**					
**10**	**M**	**4**	**46,XY,der(14;21)(q10;q10),t(6;12)(q21;q13),+21**			**x**			
**11**	**F**	**0**	**44,X,der(13;14)(q10;q10)**	**x**					
**12**	**F**	**0**	**44,XX,der(14;21)(q10;q10),der(14)t(14;22)(q32;q11.2),-22**			**x**			
**13**	**M**	**0**	**45,XY,der(21)del(21)(q22)t(21;22)(q10;q10),-22**					**x**	
**14**	**F**	**24**	**45,XX,der(21;22)(q10;q10),t(3;14)(q27;q13)**					**x**	
**15**	**F**	**26**	**45,X,der(21;22)(q10;q10),del(X)(q21)**					**x**	
**16**	**M**	**26**	**46,XXY,der(13;14)(q10;q10)**	**x**					
**17**	**F**	**27**	**mos 46,XXX,der(13;14)(q10;q10)[32]/44,X,der(13;14)(q10;q10)[28]**	**x**					
**18**	**M**	**27**	**46,XXY,der(13;14)(q10;q10)**	**x**					
**19**	**F**	**0**	**mos 45,XX,der(21;21)(q10;q10)[56]/46,XX,der(21;21)(q10;q10)[4]**				**x**		
**20**	**M**	**0**	**mos 46,XY,der(21;21)(q10;q10)[14]/46,XY[46]**				**x**		
**21**	**M**	**34**	**mos 45,XY,der(21;22)(q10;q10)[56]/46,XY,der(21;22)(q10;q10),+21[4]**					**x**	
**22**	**F**	**0**	**mos 46,XX,der(21;21)(q10;q10)[16]/46,XX[44]**				**x**		
**23**	**F**	**21**	**mos 45,XX,der(22;22)(q10;q10)[23]/46,XX[37]**						**x**
**24**	**F**	**22**	**mos 45,XX,der(14;21)(q10;q10)[9]/46,XX[51]**			**x**			
**25**	**F**	**33**	**mos 45,XX,der(21;21)(q10q10)[41]/46,XX[19]**				**x**		

### Nonrandom distributions of different subtypes of balanced ROBs

The 583 cases with balanced ROBs can be further classified into two subgroups based on their ages: 544 (93%) adults with ages ranging from 19 to 64 years-old (Table 1A) and 39 (7%) children with ages ranging from newborn to 11 years-old (Table 1B). Of the 544 adults with balanced ROBs, 524 (96.32%) are heterologous ROBs and 20 (3.68%) are homologous ROBs. The distribution of different subtypes of ROBs in these adults is nonrandom, with der(13;14) and der(14;21) constituting 59.4% and 14.7% respectively, and all other 13 subtypes of ROBs constituting the remaining ∼26% ROBs with variable proportions from 1.65% for der(15;22) to 4.23% for der(13;15). The 20 homologous ROBs identified in this cohort include 5 der(13;13), 4 der(14;14), 2 der(15;15), 6 der(21;21), and 3 der(22;22).

Of the 39 children (20 females and 19 males) with balanced ROBs, 36 (92.3%) of them are heterologous and only 3 (7.7%) of them are homologous and all of the 3 homologous ROBs are der(15;15). Similar to what observed in the adults with balanced ROBs, the distributions of different subtypes of ROBs in these children are nonrandom, with der(13q14q) and der(14q21q) being predominant, constituting 54% and 15% respectively, and all others constituting the remaining ∼30%.

### Significantly skewed male/female ratios in individuals with balanced ROBs

The male/female ratio of the 544 adults with balanced ROBs is 0.72 (227 males/317 females) (Table 1B), showing a significant difference from the male/female ratio of 0.93 for the total cases analyzed in this study (98,686 males and 105,995 females) (p<0.01). The adjusted male/female ratio in these balanced ROB carriers is 0.77(0.72/0.93), showing an excess of females being a carrier of balanced ROBs. It was noticed that all 6 der(21;21) carriers are females.

Different from 0.77 of the adjusted male/female ratio in the adults, the adjusted male/female ratio (1.02) was not skewed in the 39 children with balanced ROBs (p>0.5) (Table 1C). Increased male/female ratio (1.75) in the der(13;14) subcategory is interesting, although statistically insignificant (p>0.10).

### Nonrandom distributions of different subtypes of unbalanced ROBs

259 of the 264 individuals with unbalanced ROBs are children with ages ranging from newborn to 12 years-old (Table 2). The remaining 5 individuals are all females referred for chromosome analysis because of infertility. Of the 264 individuals with unbalanced ROBs, almost all of them are unbalanced ROBs involving chromosome 21 except a single ROB with [46,XX,der(13;14),+13] identified in a newborn girl with multiple congenital anomalies (S2a Fig). The distribution of different subtypes of unbalanced ROBs in these children are nonrandom, 47.5% for der(21;21), 41.7% for der(14;21), 6.6% for der(13;21), and 2.3% for both der(15;21) and der(21;22) respectively.

### Male/female ratio in individuals with unbalanced ROBs

Of the 259 children with unbalanced ROBs, the male/female ratio is 1.09 (135/124), when adjusted, 1.17 (1.09/0.93). Although more males are observed in this group, statistically the male/female ratio is not significantly increased (p>0.10).

### Mosaic ROBs

The 9 mosaic ROBs in Table 3 are composed of 3 balanced (Cases 23, 24, and 25), 4 unbalanced der(21;21) (Cases 20, 21, and 22), and 2 unbalanced being both mosaic and complex (case 4 identified in a newborn boy with DS and case 17 identified in a 27 years-old female with atypical features of Turner syndrome). Four of the 9 mosaic ROBs were identified in children (2 males and 2 females) containing unbalanced der(21;21), and the remaining 5 were identified in adults (1 male and 4 females).

### Complex ROBs

There are 18 complex ROBs including 2 mosaic ROBs mentioned above (cases 4 and 17), and 16 cases in non-mosaic status. In total, 13 cases were observed in children and 5 cases in adults.

The 13 complex ROBs in children include: (1) one ROB of trisomy 18 with concurrent existence of non-contributory der(14;21) [46,der(14;21),+18] (case 1), (2) seven ROBs being standard trisomy 21 with concurrent existence of non-contributory der(13;14) [46,der(13;14),+21] (cases 2, 3, 5–9), (3) one ROB trisomy 21 from contributory der(14;21) with concurrent existence of a reciprocal translocation between chromosomes 6 and 12 (case 10), (4) one ROB with loss of a chromosome X with concurrent existence of der(13;14) [44,X,der(13;14)] (case 11), (5) one ROB with concurrent existence of a balanced der(14;21) and a der(14) from a translocation between chromosomes 14 and 22 (case 12), (6) one ROB of der(21;22) with a distal deletion on the der(21;21)(case 13), and (7) the mosaic ROB containing an unbalanced der(21;21) in one cell population and a balanced der(13;21) in the other cell population (case 4).

The 5 complex ROBs in adults include one ROB with concurrent existence of der(21;22) and a reciprocal translocation between chromosomes 3 and 14 (case 14), one ROB with concurrent existence of der(21;22) and a deletion on one of chromosomes X (case 15), two ROBs of Klinefelter syndrome with concurrent existence of non-contributory der(13;14) [46,XXY,der(13;14)] (cases 16 and 18), and the mosaic one containing two abnormal cell populations, one has three copies of chromosome X and the other has a single copy of chromosome X, and both populations contain a balanced der(13;21) (case 17).

## Discussion

### Balanced ROB carriers

It is well known that balanced ROB carriers usually have normal phenotype, but can have problem of infertility associated with oligospermia in male adults, miscarriage or infertility in female adults. Although rare, a variety of abnormal phenotypes were described in some balanced ROB carriers [13–16]. Warburton et al estimated that approximately 3.7% of *de novo*, balanced ROBs resulted in abnormal phenotypes [16]. Proposed explanations for the abnormal phenotypes in these balanced ROB carriers include (1) mosaicism arising from postmeiotic trisomy rescue mainly for heterologous ROBs and monosomy rescue mainly for homologous ROBs, and (2) aberrant genomic imprinting involving chromosomes 14 and 15, and (3) homozygosity of autosomal recessively inherited mutations [2,17].

To our knowledge, the current study reported the largest collection of balanced ROBs (in total 583 balanced ROBs including 544 adults and 39 children). The referral indications for the 39 children (36 heterologous ROBs and 3 homologous ROBs) were variable. Since we don’t have either complete clinical data or thorough genetic/genomic testing information, such as methylation analysis, we are uncertain whether the balanced ROBs in these children were the underlying causes for their clinical features. We postulate that the majority of the 36 heterologous ROBs are likely to be incidental findings for following reasons: (1) clinical referral indications were diverse within each of the subcategories, for example, among the 21 children with der(13;14) or the 6 children with der(14;21); (2) the risk to be UPD due to ROBs involving chromosomes 14 or 15 is estimated to be less than 1% [13,17], (3) the risk for “isozygosity” for recessive gene is likely to be very low since none of the common recessive genes suitable for population screening has its locus on an acrocentric chromosome [18]. In contrast, we postulate that the der(15;15) present in the 3 newborns might be responsible for their clinical features given the fact that the majority of homologous ROBs including der(15;15) are UPD due to the formation of isochromosomes arising postzygotically [3].

The referral reasons for all of the 544 balanced adult ROBs (524 heterologous ROBs and 20 homologous ROBs) are mainly because of infertility, miscarriage, or offspring(s) with known ROB abnormalities, consistent with previous findings that balanced ROBs were enriched in individuals with infertile problems [17,19–22]. In addition to their infertile problems, we postulate that approximately 3 to 4 individuals out of the 524 heterologous ROB carriers might have imprinting disorders related to UPD(14) or UPD(15) based on the estimation by Berend et al that 0.8% of heterologous ROB carriers are likely to be UPD [5,13,17]. Different from these heterologous ROB carriers, the majority of the 20 homologous ROBs are likely to be UPD due to isochromosomes arising postmeiotically, and the 6 of the 20 balanced homologous ROBs [4 der(14;14) and 2 der(15;15)] were likely to have additional clinical features related imprinting disorders except their reproductive difficulties.

Of the 544 adults ROB carriers with reproductive difficulties, 96.32% is heterologous ROBs and 3.68% is homologous ROBs. These results is significantly different from the data in infertile Caucasian ROB carriers reported by Therman et al in which 90% of them were heterologous and 10% of them were homologous (P<0.01) [5]. Nonrandom distribution of balanced ROB subcategories were observed in all relevant studies [2,5] including ours, for example, der(13;14) and der(14;21) constitute 59.4% and 14.7% respectively in the 544 adults ROB carriers with reproductive difficulties observed in this study. We noticed that the percentage of der(13;14) is significantly decreased and the percentage of der(14;21) is significantly increased in the current study when compared with the data reported by Therman et al [5] or with the accumulated data reviewed by Gardner et al in which the proportions of der(13;14) and der(14;21) were 74% and 8% respectively [2]. Since there is no available data showing the distribution of balanced ROBs in Chinese population, we are not sure whether these differences between Chinese and Caucasian with infertile difficulties are due to ethnic difference or any other factors. Given the facts that almost all of the balanced ROB carriers are phenotypically normal and remain undetected until some of them experience difficulties to reproduce, the distributions of balanced ROBs in this cohort might not have significant difference from their distributions in general population although the balanced ROB carriers were highly concentrated in individuals experiencing reproductive difficulties.

Excessive females were observed in the 544 adults balanced ROBs with the adjusted male/female ratio of 0.77 (P<0.01) (Table 1B). This phenomenon was observed previously for both prenatal and postnatal cases and possible reason for the female predominance is primarily explained by infertility difficulties in male carriers [23,24]. The adjusted male/female ratio (1.02) in the 39 children is not skewed (Table 1C), which is different from the 544 adults where excessive females were observed. The difference could be explained by different referral reasons between adults and children requested for chromosomal analysis. When the male/female ratios in different subcategories of the adult ROBs were further stratified, the adjusted male/female ratio in der(13;14) subcategory is 1.02, showing similar numbers of males and females in this subgroup while there are more females in other subcategories although the sample sizes are too small to reach statistical differences (Table 1B). Consistent with the trend observed in adults, it is interesting to notice that more males are present in the subcategory of children with der(13;14), indicating that different formation mechanisms might differentiate der(13;14) subcategory from other ROB subcategories as proposed by Bandyopadhyay et al [6,7].

### Unbalanced ROBs

Except a single ROB of trisomy 13 observed in a newborn girl with a karyotype of [46,XX,der(13;14),+13] (S2a Fig), all remaining 263 of the 264 unbalanced ROBs identified in this study were ROB of trisomy 21 (Table 2), a finding consistent with known conclusions that, only trisomies 21 and 13 out of the acrocentric chromosomes are viable in the newborn. The 263 unbalanced rob(21) accounted for about 4.6% of the 5,772 individuals with DS (our unpublished data) which is slightly higher than the 4.1% from a nationwide population-based study in Denmark and significantly higher than the 3.3% from the U.S. population-based birth defects registries [25,26]. Although ascertainments might have caused the differences, ethnic factor cannot be excluded [27]. We further compared our results with the data reported by Mutton et al in which they reported the largest systematic collections of data from a single source about the cytogenetic and epidemiological findings in DS in England and Wales from 1989 to 2009 [9]. They identified a total 779 ROBs with translational trisomy 21 including 338 (43.4%) der(21;21) and 441 (56.6%) other subtypes of ROBs. There are no significant differences about the proportions of different subtypes of unbalanced ROBs between our data and theirs.

Of the 263 non-mosaic unbalanced ROB trisomy 21, 5 of them were adult females and all remaining 258 were children. All the 5 adult females were living in the countryside and were referred for chromosome analysis because of infertility. We presume that reasons for these 5 female individuals being referred for chromosome analysis until they were noticed to have infertile problems are more likely because of economic-social situations rather than atypical clinical features of trisomy 21.

Excluding the 5 adult females, the adjusted male/female ratio of the 258 children with ROB trisomy 21 is 1.17, showing slight higher male proportion in this group. Our data is similar to the 1.14 of ROB trisomy 21 from the New York State Chromosome Registry on over 10,000 DS reported from 1977 to 1996 [28], and is also similar to the report by Mutton et al in which 51.6% were male among der(21;21) and 55.1% were male among other subtypes of unbalanced ROBs [9]. The genetic mechanisms leading to excessive male probands of DS are unknown. Griffin et al. proposed that some of the excess of males among DS individuals is attributable to a nondisjunctional mechanism in which the extra chromosome 21 preferentially segregates with the Y chromosome [29]. When comparing the distribution of different subtypes of unbalanced ROBs between the current study with the two prestigious reports mentioned above, similar proportions were noticed indicating that there are no significant differences about the distribution of different subtypes of unbalanced ROBs between Caucasians and Chinese [9,28].

### Mosaic ROBs

Postnatal mosaic ROBs are rare [24,30,31]. Nine mosaic ROBs (6 females and 3 males) were identified in the current study, consistent with previous reports that excess mosaic females were obsevered on individuals with autosomal abnormalities [24,28,32]. Proposed explanations for this phenomenon include specific chromosome loss in females and an increased rate of rescue of trisomy to disomy in female conceptions [24,33]. The formation of these mosaic ROBs occurred mainly postzygotically, somehow different from non-mosaic ROBs which usually occurred during meiosis [2,3,13]. It was also noticed that different subtypes of mosaic ROBs occurred through different multiple-step mechanisms [30,31,34]. Mosaicism with more than one ROBs are extremely rare [24]. The karyotype of [45,XY,der(13;21)/46,XY,der(21;21)] identified in this study has not been reported although two relevant ROBs with possibly similar mechanisms were documented with karyotype of [45,XY,rob(13;21) /46,XY,rea(21;21), +21/46,XY] [35,36]. Possible mechanisms leading to the formation of the mosaicism with more than one ROBs was proposed by Bandyopadhyay et al [30]. In brief, a sperm containing a normal chromosome 13 and 21 fertilized an egg containing normal chromosome 13 and 21, resulting in a normal conceptus. Due to ‘‘instability” of the paternally inherited chromosome 21, two independent rearrangement events occurred, resulting in two different cell lines, the balanced rob(13q21q) cell line formed between the maternal chromosome 14 and the paternal chromosome 21 and the trisomy 21 cell line containing an isochromosome 21 formed from the paternally inherited chromosome 21.

### Complex ROBs

In the current study, we reported 18 cases with multiple chromosomal abnormalities with at least one of them involving an acrocentric chromosome. To our knowledge, except [46,der(13;14),+21] (S2b Fig), [44,X,der(13;14)] (S2c Fig) and [46,XXY,der(13;14)] (S2d Fig) have been observed previously [9,10], the remaining complex ROB karyotypes have not been reported yet (S8b and S14 Figs). We noticed that trisomy 18 with an additional balanced *de novo* der(13;14) was previously described in two cases, which was similar to the [46,der(14;21),+18] observed in our study [37,38]. It is not surprised to notice that all the individuals with complex ROBs who could survive to birth were these with ROBs containing reciprocal translocations or ROBs with double aneuploidies involving chromosomes X, Y and 21. It is also interesting to notice that the 13 children with complex ROBs have more deleterious karyotypes than that in the 5 adults. These quite uncommon observations involving multiple chromosomal abnormalities could be explained as random events or more likely caused by mitotic interchromosomal effect that enhances genetic instability during early development of embryos of Robertsonian translocation carriers [39].

### Weaknesses of this study

There are several weaknesses present in this study: (1) bias of ascertainments; (2) incomplete clinical information about these individual carrying ROBs; and (3) incomplete follow-up molecular testing, especially for these ROBs with homologous ROBs. Although we think these weaknesses do not affect the result trends observed in this study, the authors cannot rule out the possibility of other ROBs present in the population that were not identified (due to selection bias) and that the clinical impact of the novel ROBs cannot be determined (selection bias and lack of associated clinical data).

## Supporting Information

S1 Fig A der(13;13) and two copies of chromosomes 14, 15, 21 and 22. (TIF)

S2 Fig A der(13;14), and (a) a single copy of chromosomes 14, and two copies of chromosomes 13, 15, 21 and 22, (b) a single copy of chromosomes 13 and 14, two copies of chromosomes 15 and 22, and three copies of chromosome 21, (c) a single copy of chromosomes X and 14, and two copies of chromosomes 15, 21 and 22, (d) a single copy of chromosomes Y, 13 and 14, and two copies of chromosomes X, 15, 21 and 22. (TIF)

S3 Fig A der(13;15), a single copy of chromosomes 13 and 14, and two copies of chromosomes 14, 21 and 22. (TIF)

S4 Fig A der(13;21), and (a) a single copy of chromosomes 13 and 21, and two copies of chromosomes 14, 15 and 22, (b) a single copy of chromosome 13, and two copies of chromosomes 14, 15, 21 and 22. (TIF)

S5 Fig A der(13;22), a single copy of chromosomes 13 and 22, and two copies of chromosomes 14, 15 and 21. (TIF)

S6 Fig A der(14;14) and two copies of chromosomes 13, 15, 21 and 22. (TIF)

S7 Fig A der(14;15), a single copy of chromosomes 14 and 15, and two copies of chromosomes 13, 21 and 22. (TIF)

S8 Fig A der(14;21), and (a) a single copy of chromosome 14, and two copies of chromosomes 13, 15, 21 and 22, (b) a der(14;22), a single copy of chromosomes 21 and 22, and two copies of chromosomes 13 and 15. (TIF)

S9 Fig A der(14;22), a single copy of chromosomes 14 and 22, and two copies of chromosomes 13, 15 and 21. (TIF)

S10 Fig A der(15;15) and two copies of chromosomes 13, 14, 21 and 22. (TIF)

S11 Fig A der(15;21), and (a) a single copy of chromosomes 15 and 21, and two copies of chromosomes 13, 14 and 22, (b) a single copy of chromosomes 15, and two copies of chromosomes 13, 14, 21 and 22. (TIF)

S12 Fig A der(15;22), a single copy of chromosomes 15 and 22, and two copies of chromosomes 13, 14 and 21. (TIF)

S13 Fig A der(21;21), a single copy of chromosome 21, and two copies of chromosomes 13, 14, 15 and 22. (TIF)

S14 Fig A der(21;22), apparently balanced translocations between chromosomes 3 and 14 [t(3;14)(q27;q13)], a single copy of chromosomes 3, 14, 21 and 22, and two copies of chromosomes 13 and 15. (TIF)

S15 Fig A der(22;22) and two copies of chromosomes 13, 14, 15 and 21. (TIF)
