# Supplementary figures and images for: Robertsonian Translocations: An Overview of 872 Robertsonian Translocations Identified in a Diagnostic Laboratory in China
Source: PLoS One. 2015 May 1;10(5):e0122647. doi: 10.1371/journal.pone.0122647 (PMC4416705; doi:10.1371/journal.pone.0122647)

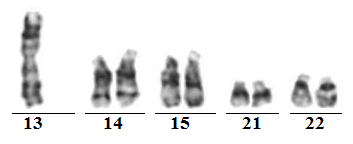

Supplement: S1 Fig — (TIF) [file pone.0122647.s001.tif]

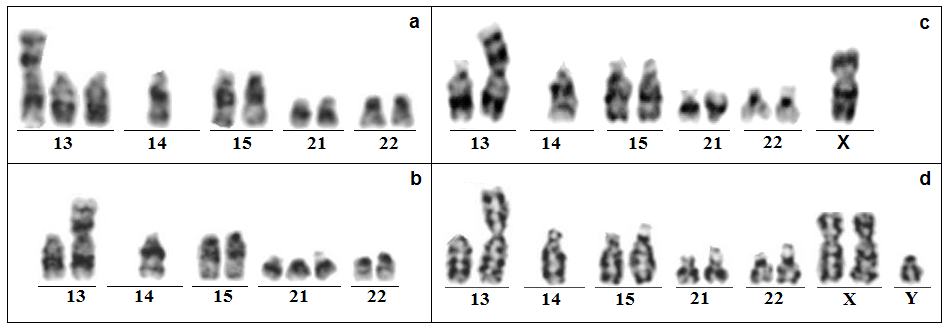

Supplement: S2 Fig — (TIF) [file pone.0122647.s002.tif]

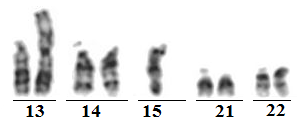

Supplement: S3 Fig — (TIF) [file pone.0122647.s003.tif]

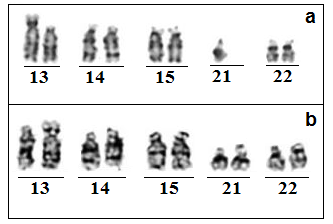

Supplement: S4 Fig — (TIF) [file pone.0122647.s004.tif]

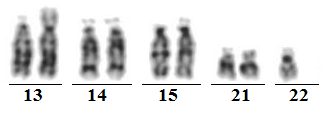

Supplement: S5 Fig — (TIF) [file pone.0122647.s005.tif]

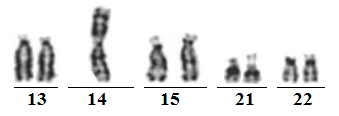

Supplement: S6 Fig — (TIF) [file pone.0122647.s006.tif]

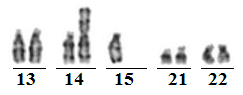

Supplement: S7 Fig — (TIF) [file pone.0122647.s007.tif]

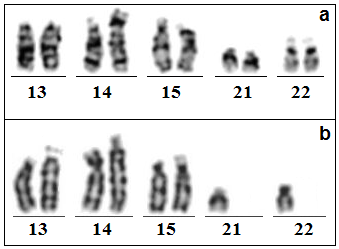

Supplement: S8 Fig — (TIF) [file pone.0122647.s008.tif]

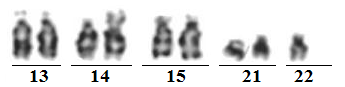

Supplement: S9 Fig — (TIF) [file pone.0122647.s009.tif]

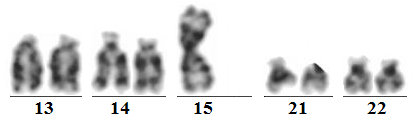

Supplement: S10 Fig — (TIF) [file pone.0122647.s010.tif]

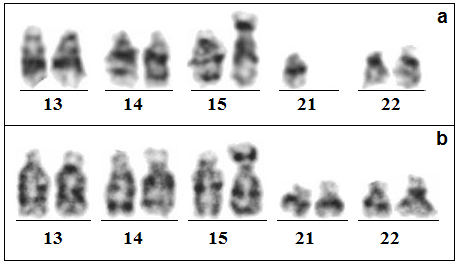

Supplement: S11 Fig — (TIF) [file pone.0122647.s011.tif]

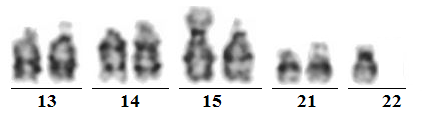

Supplement: S12 Fig — (TIF) [file pone.0122647.s012.tif]

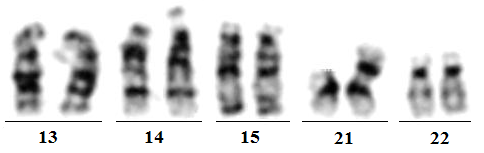

Supplement: S13 Fig — (TIF) [file pone.0122647.s013.tif]

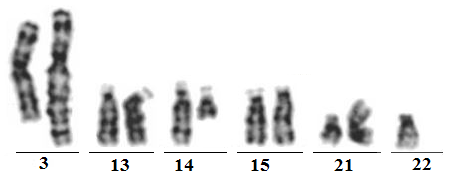

Supplement: S14 Fig — (TIF) [file pone.0122647.s014.tif]

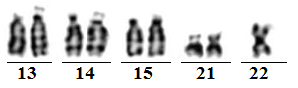

Supplement: S15 Fig — (TIF) [file pone.0122647.s015.tif]
